# Associations Between Physical Capability Markers and Risk of Coronary Artery Disease: A Prospective Study of 439,295 UK Biobank Participants

**DOI:** 10.3390/healthcare13091018

**Published:** 2025-04-28

**Authors:** Duqiu Liu, Chenxing Yang, Tianyu Guo, Yi Guo, Jinjie Xiong, Ru Chen, Shan Deng

**Affiliations:** 1 Clinic Center of Human Gene Research, Union Hospital, Tongji Medical College, Huazhong University of Science and Technology, Wuhan 430022, China; drliuduqiu@163.com (D.L.); hxshuyun@hotmail.com (Y.G.); 2 Liyuan Cardiovascular Center, Tongji Medical College, Huazhong University of Science and Technology, Wuhan 430077, China; 3 Department of Epidemiology and Biostatistics, School of Public Health, Tongji Medical College, Huazhong University of Science and Technology, Wuhan 430030, China; bmohufbmpi@163.com; 4 Department of Nutrition and Food Hygiene, Hubei Key Laboratory of Food Nutrition and Safety, Ministry of Education Key Laboratory of Environment and Health, and State Key Laboratory of Environment Health (Incubating), School of Public Health, Tongji Medical College, Huazhong University of Science and Technology, Wuhan 430030, China; 18210047875@163.com; 5 Department of Cardiology, Union Hospital, Tongji Medical College, Huazhong University of Science and Technology, Wuhan 430022, China; xiong_jingjie@163.com

**Keywords:** sarcopenia, grip strength, muscle mass, coronary artery disease, polygenic risk score

## Abstract

**Background**: The relationship between sarcopenia and the incidence of coronary artery disease (CAD) is not well understood. This study aimed to investigate this relationship and the modifying effect of potential risk factors. **Methods**: We conducted a prospective study including 439,295 individuals from the UK Biobank. The primary outcome was the incidence of CAD. The main physical capability markers for sarcopenia, grip strength and muscle mass, were investigated as risk factors of interest. Grip strength was measured using a Jamar J00105 (Lafayette, IN, USA) hydraulic hand dynamometer, while muscle mass was estimated through bioelectrical impedance. Cox proportional hazard models were employed to analyze the associations between the exposures and the risk of CAD. **Results**: A total of 41,564 incident cases of CAD were identified after a median follow-up of 13.15 years (IQR 12.29–13.88 years). Compared with the lowest quintile of grip strength, the adjusted HRs for incidences of CAD from the second to the fifth quintile were 0.81 (95% CI: 0.79–0.83), 0.71 (95% CI: 0.69–0.73), 0.61 (95% CI: 0.60–0.63), and 0.49 (95% CI: 0.48–0.51). The association remained significant in subgroup analysis and interactions were observed between the two exposures and sex, age, smoking status, inflammatory diseases, metabolic syndrome, and genetic predisposition (all *p* for interactions < 0.05). **Conclusions**: Physical capability markers of sarcopenia, grip strength and muscle mass, were independently associated with a dose–response decreased risk for CAD incidence, regardless of genetic predisposition and potential modifying risk factors.

## 1. Introduction

Coronary artery disease (CAD) is a significant public health concern that contributes to a substantial loss of health and resultant social burden [1]. CAD is the main component and cause of ischemic heart disease (IHD), which accounted for approximately 9.4 million cardiovascular deaths and 1850 disability-adjusted life years per 100,000 globally in 2021 [2]. Management of non-modifiable (age, sex, ethnicity, and genetic background) and modifiable (related to environment, metabolism, and behavior) risk factors for CAD play a crucial role in the primary and secondary prevention of CAD [3,4,5,6]. Despite advancements in understanding of cardiovascular disease (CVD) and its risk factors, the absolute number of incident and prevalent cases of IHD and the overall burden of disability-adjusted life years (DALYs) attributed to IHD, have continued to rise since 1990 [7]. Although global age-standardized rates for prevalent cases, deaths, and DALYs of IHD have declined from 1990 to 2019, regions in South, East, and Southeastern Asia still experience an increasing age-standardized death rate [7]. Given the global trends of population growth and aging, there is an ongoing need to comprehensively identify risk factors associated with CAD.

Sarcopenia is a progressive skeletal muscle disorder characterized by the loss of muscle strength and muscle mass [8]. While it is most commonly observed in the elderly population, it can also occur in middle-aged individuals with underlying health conditions [9]. The reported incidence of sarcopenia is 1.6% in European individuals aged 40–79 years [10], 3.6% in English people aged 85 years [11], and 3.4% in a group of Chinese adults [12]. In patients with CVDs, the prevalence of sarcopenia has been reported to be twice as high compared to the general population [13]. Several cardiovascular conditions, such as coronary atherosclerosis, myocardial infarction, coronary artery calcification (CAC), and atrial fibrillation, have been found to be associated with sarcopenia in cross-sectional studies [14,15,16]. Longitudinal studies have established that individuals with low skeletal muscle mass were at an elevated risk for CAC, major adverse cardiovascular events (MACEs), and all-cause mortality among patients with CAD [17,18,19]. Decreased muscle strength has also been reported to be associated with the risk of CAD [20,21].

Despite the accumulating evidence on the association between sarcopenia and CAD, it the shape of the association remained unclear. Prospective studies with long-term follow-up were lacking. The interactions between muscle mass, grip strength, and other potential cardiovascular risk factors in CAD were unknown. Genetic factors are important in the risk assessment of CAD [22]. However, to our knowledge, no studies have examined the interaction between sarcopenia and genetic predisposition on CAD. To address these gaps, we comprehensively examined the association between physical capability markers for sarcopenia and CAD, as well as the potential modifying risk factors, by utilizing data from the United Kingdom (UK) Biobank cohort.

There was limited evidence from large prospective studies regarding the association between sarcopenia and coronary artery disease (CAD). Thus, this study aimed to investigate the relationships between physical capability markers and the incidence of CAD as well as the modifying effect of potential risk factors.

## 2. Methods

### 2.1. Study Population

UK Biobank is a prospective cohort that recruited more than 500,000 participants aged 40–69 from 22 assessment centers throughout the UK during 2006–2010 [23]. Each participant provided electronic signed consent and completed a self-completed touch-screen questionnaire, physical and functional measurements, and computer-assisted interview. Additionally, biological samples including blood, urine, and saliva were collected at baseline [24]. Follow-up data were obtained through linkage to a range of national datasets [24]. The ethical approval for UK Biobank was obtained from the North West Multi-Centre Research Ethics Committee (21/NW/0157). Comprehensive details about UK Biobank can be found online (www.ukbiobank.ac.uk). Data used in this work were extracted under access application number 109,546. In this study, 502,356 individuals with follow-up data at baseline were included. Participants with CAD (n = 12,733) or cancer (n = 13,889) at baseline, incomplete data for the exposures (n = 15,740), and missing data for covariates (n = 16,114) were excluded. Participants who developed CAD within the first 2 years of follow-up (n = 4585) were also excluded to minimize the effect of reverse causality. A flow diagram illustrating the recruiting process is available in Figure S1.

### 2.2. Assessment of Sarcopenia

Sarcopenia was defined by low muscle strength, muscle mass, and physical performance [25]. Therefore, grip strength, muscle mass, and gate speed were selected as physical capability markers for sarcopenia [26].

A Jamar 100,105 hydraulic hand dynamometer was used to measure grip strength, following the methodology described in the previous literature [27]. Briefly, both hands were measured in turn and the recorded values were expressed in absolute units (kilograms, kg). The decision to use absolute terms for grip strength was based on evidence showing that expressing grip strength in absolute or relative terms did not affect its association with health outcomes [28]. The mean value of grip strength from both hands was used for subsequent analyses.

Muscle mass was assessed through bioelectrical impedance analysis (BIA, Tanita BC418MA Body Fat Analyser, Tanita, Tokyo, Japan). Before the assessment, participants completed a clinical examination that included measurements of grip strength, waist and hip circumference, weight, and standing height. BIA was not feasible for participants who were wheelchair-bound or unable to stand. After obtaining measurements of total body composition by BIA, skeletal muscle mass was then estimated using the Janssen equation [18]. To eliminate the influence of body size on the accuracy of BIA, muscle mass was divided by body weight.

Since the UK biobank does not provide an objective measurement for gait speed, self-reported walking pace was utilized as a proxy for this variable in sensitivity analysis and divided into categories of slow, average, or brisk.

### 2.3. Outcome

The outcome in this study was the incidence of CAD. The occurrence of CAD was identified through linkage with various sources, including death register, primary care records, and hospital inpatient records. CAD was defined based on hospital procedure codes, electronic health, and self-reported information (Table S2). Hospital inpatient admission data for different regions in the United Kingdom were obtained from specific sources. For England, the data were provided by the Data Access Request Service (DARS), which is managed by National Health Service (NHS) in England. In Wales, the data were sourced from the Secure Anonymised Information Linkage (SAIL) Databank, located at the University of Swansea, and managed by NHS Wales Informatics Service’s Information Services Division (ISD). For Scotland, the data were obtained from Public Health Scotland, which is a part of NHS National Services Scotland (NSS).

### 2.4. Covariates

Sex, ethnicity, and qualification (college or university, or others) were self-reported by the participants. Age was determined based on the date of birth at baseline. Deprivation was derived by the Townsend score [26]. The smoking status of the participants was self-reported and classified into three categories: never, previous, or current smoker. Physical activity was determined by administering the International Physical Activity Questionnaire—Short Form. A cumulative score for dietary risk factors was calculated using 9 food items according to the current guidelines in the UK [29]. A lower score on this scale (range from 0 to 9) indicated a healthier diet. Body mass index (BMI) was computed by dividing the weight in kilograms by the square of the height in meters. Inflammatory diseases (arthritis, inflammatory bowel disease, and asthma) were self-reported at baseline [30]. Metabolic syndrome was diagnosed in individuals with any three of its components (central obesity, high glycaemia/diabetes, high blood pressure/hypertension, low HDL, and high triglyceride) [31,32]. Central obesity was defined as an enlarged waist circumference (≥88 cm in women and ≥102 cm in men) [32]. High glycaemia/diabetes was defined as a fasting glucose level exceeding 5.6 mmol/L or a previous history of diabetes [31,32]. High blood pressure/hypertension was diagnosed if the baseline blood pressure was recorded as 130/85 mmHg or higher, or if there was a documented history of hypertension [31,32]. Dyslipidemia encompassed elevated triglyceride levels surpassing 1.7 mmol/L and reduced levels of high-density lipoprotein cholesterol (HDL-C), which were below 1.3 mmol/L in women and below 1.0 mmol/L in men [31,32].

### 2.5. Genetic Risk Score for Coronary Artery Disease

Polygenic risk scores (PRS) for CAD were obtained from Standard PRS (Category 301) provided by UK biobank. The UK Biobank has provided comprehensive information on deep genotyping and genomic data [33]. The PRS for CAD were created for all individuals in the UK biobank by meta-analyzing multiple external genome-wide association study (GWAS) sources [34].

### 2.6. Statistical Analysis

Baseline characteristics were presented as mean with standard deviation (SD) for quantitative data and numbers (percentages) for categorical data.

The relationships between grip strength, muscle mass, and CAD were estimated using Cox proportional hazard models by quintiles of exposures, with the lowest quintile serving as the reference group. A test for trend was performed based on a variable containing a median value for each quintile. The non-linear associations between grip strength, muscle mass, and CAD were investigated by restricted cubic spline (RCS) fitted in Cox proportional hazard models, and the average value of each exposure was selected as the reference group. The proportional hazard assumption was tested using the Schoenfeld residual method.

The analyses were adjusted for the aforementioned covariates including age, sex, qualifications, deprivation, ethnicity, smoking, physical activity, diet score, physical activity, BMI, inflammatory diseases, and metabolic syndrome (central obesity, high glycaemia/diabetes, high blood pressure/hypertension, low HDL, and high triglyceride). The relationship between the exposures, covariates, and the outcome was depicted in Figure S2. Several sensitivity analyses were performed: (i) investigating the relationship between Gait speed and CAD; (ii) excluding people with extreme values (±2.5 standard deviation [SD] from the median).

Finally, subgroup analyses were performed by stratifications based on potential risk factors including sex, age, qualification, smoking status, physical activity, BMI, inflammatory diseases, and metabolic syndrome. Stratified analysis by CAD genetic risk was also conducted and genetic predisposition was categorized into low genetic risk (quartile 1), intermediate genetic risk (quartile 2 and 3), and high genetic risk (quartile 4), based on PRS.

SAS 9.4 (SAS Institute Inc., Cary, NC, USA) and R version 4.3.1 (R Foundation for Statistical Computing, Vienna, Austria) were used for data analysis. A two-sided *p* ≤ 0.05 was considered statistically significant.

## 3. Results

At baseline, a total of 502,356 participants were included in this prospective study. After excluding people with previous CAD, cancer, and missing or incomplete data, the final analysis included 439,295 participants (Figure S1). After a median follow-up of 13.15 years (interquartile range [IQR] 12.29–13.88 years), 41,564 individuals experienced CAD events, including 39,466 hospitalizations and 2098 deaths.

Baseline characteristics of the population are presented in Table 1. Most of the participants were white (94.5%), with a mean age of 56.73 years at baseline, and 45.1% were male. In total, 66.9% of the participants received a college or university degree and 55.5% of them were non-smokers. Inflammatory diseases (arthritis, inflammatory bowel disease, and asthma) and metabolic syndromes were noted in 14.9% and 29.1% of the population, respectively. Participants with higher grip strength or muscle mass were generally younger and more physically active. In comparison to those in the lowest quintile, individuals in the higher quintiles of grip strength or muscle mass were less likely have comorbidities of inflammatory diseases and components of metabolic syndromes.

**Figure 1:**
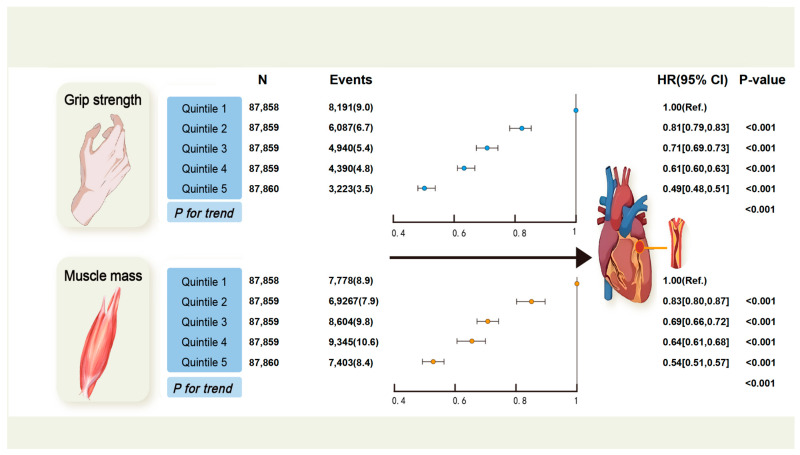
Associations between quintiles of physical capability markers and coronary artery disease. Grip strength and muscle mass were divided into quintiles. Associations between each exposure and CAD were analyzed using Cox proportional hazard models. Participants with existing CAD or cancer at baseline, or with insufficient data on the exposures or covariates, were excluded from the analyses. We adjusted the analyses for age, sex, qualification, deprivation, ethnicity, smoking, physical activity, diet score, inflammatory disease, BMI, and metabolic syndrome (central obesity, high glycaemia/diabetes, high blood pressure/hypertension, low HDL, and high triglyceride). A *p* value < 0.05 was considered statistically significant. N—number; HR—hazard ratio; CAD—coronary artery disease; BMI—body mass index; HDL—high-density lipoprotein.

The associations between quintiles of grip strength, muscle mass, and incident CAD are depicted in Figure 1. Elevated grip strength or muscle mass were associated with a decreased risk of CAD in a dose–response manner. Compared with the lowest quintile of grip strength, the adjusted HRs for incident CAD from the second to the fifth quintile were 0.81 (95% confidence interval [CI]: 0.79–0.83), 0.71 (95% CI: 0.69–0.73), 0.61 (95% CI: 0.60–0.63), and 0.49 (95% CI: 0.48–0.51), respectively (*p* for trend < 0.001). The HRs across quintiles of muscle mass were 1.00 (reference), 0.83 (95% CI: 0.80–0.87), 0.69 (95% CI: 0.66–0.72), 0.64 (95% CI: 0.61–0.68), and 0.54 (95% CI: 0.51–0.57), respectively (*p* for trend < 0.001).

**Figure 2:**
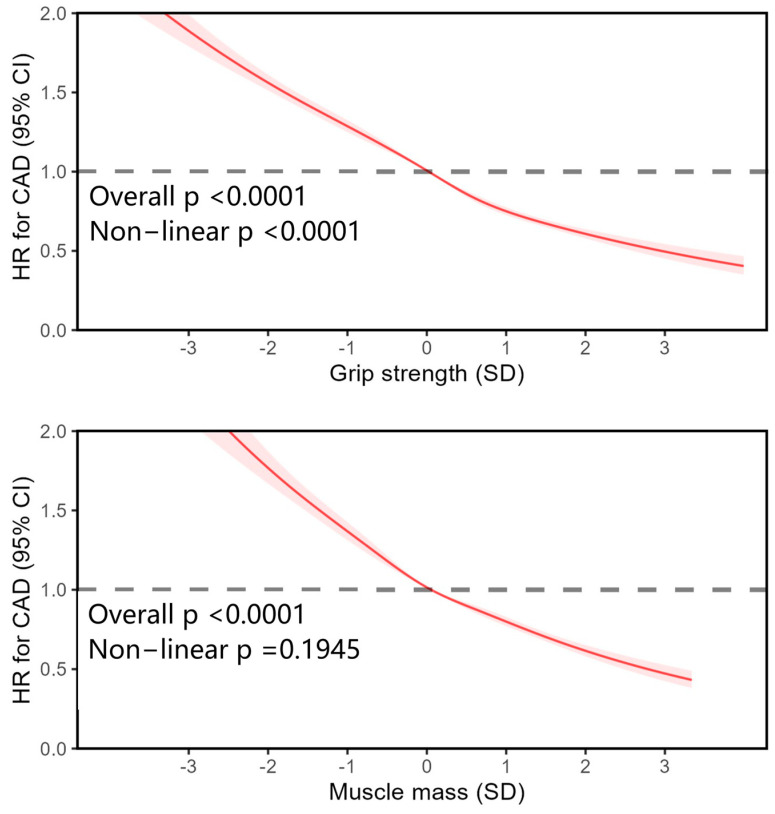
Associations between grip strength, muscle mass, and coronary artery disease. The non-linear relationships between grip strength, muscle mass, and CAD were analyzed by employing restricted cubic splines in Cox proportional hazard models. The average value of each exposure was selected as the reference group. Participants with existing CAD or cancer at baseline, or with insufficient data on the exposures or covariates, were excluded from the analyses. Potential confounders including age, sex, qualification, deprivation, ethnicity, smoking, physical activity, diet score, inflammatory disease, BMI, and metabolic syndrome (central obesity, high glycaemia/diabetes, high blood pressure/hypertension, low HDL, and high triglyceride) were adjusted in the analysis. A *p* value < 0.05 was considered statistically significant. CAD—coronary artery disease; HR—hazard ratio; BMI—body mass index; HDL—high-density lipoprotein; SD—standard deviation.

This dose–response relationship was further flexibly modeled and visualized by restricted cubic spline (RCS) (Figure 2). The association between grip strength and CAD exhibited a non-linear pattern (non-linear *p* < 0.0001), whereas the relationship between muscle mass and CAD was linear (non-linear *p* = 0.1945). Importantly, these associations remained significant after excluding individuals with extreme values (Figure S3). A sensitivity analysis on the association between gait speed, each confounder, and CAD was also performed (Figure S4). Individuals who walked at an average or brisk pace had a reduced likelihood of developing CAD compared to those with slow pace (HR_averge pace_: 0.54; 95% CI 0.52–0.55 and HR_brisk pace_: 0.39; 95% CI 0.38–0.41).

**Figure 3:**
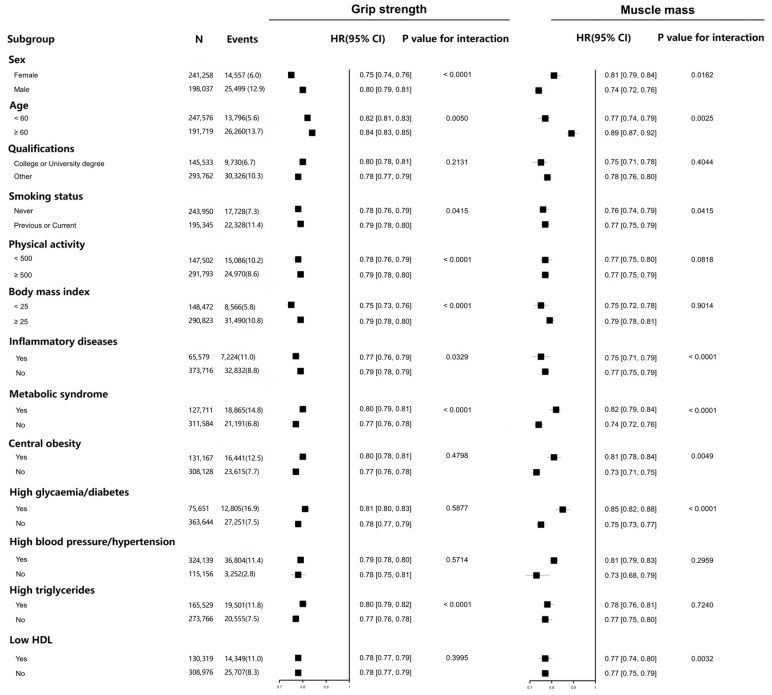
Association of grip strength, muscle mass, and the risk of incident CAD stratified by potential risk factors. Grip strength and muscle mass were divided into quintiles. Associations between each exposure and CAD in each subgroup were analyzed using Cox proportional hazard models. Participants with existing CAD or cancer at baseline, or with insufficient data on the exposures or covariates, were excluded from the analyses. We adjusted the analyses for age, sex, qualification, deprivation, ethnicity, smoking, physical activity, diet score, inflammatory disease, BMI, and metabolic syndrome (central obesity, high glycaemia/diabetes, high blood pressure/hypertension, low HDL, and high triglyceride). A *p* value < 0.05 was considered statistically significant. N—number; HR—hazard ratio; CAD—coronary artery disease; BMI—body mass index; HDL—high-density lipoprotein.

**Figure 4:**
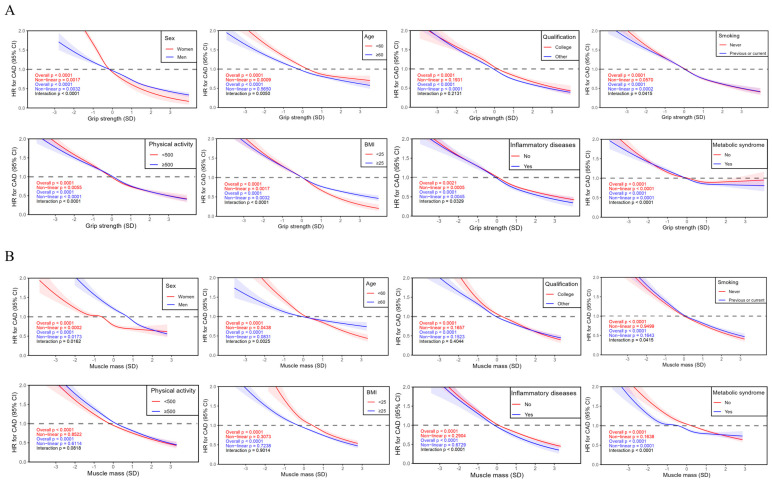
Association between grip strength, muscle mass, and coronary artery disease by subgroup. (**A**) The association between grip strength and CAD by subgroup. (**B**) The association between muscle mass and CAD by subgroup. The non-linear relationships between grip strength, muscle mass, and CAD in each subgroup were analyzed by employing restricted cubic splines in Cox proportional hazard models. The average value of each exposure was selected as the reference group. Participants with existing CAD or cancer at baseline, or with insufficient data on the exposures or covariates, were excluded from the analyses. Potential confounders including age, sex, qualification, deprivation, ethnicity, smoking, physical activity, diet score, inflammatory disease, BMI, and metabolic syndrome (central obesity, high glycaemia/diabetes, high blood pressure/hypertension, low HDL, and high triglyceride) were adjusted in the analysis. A *p* value < 0.05 was considered statistically significant. CAD—coronary artery disease; HR—hazard ratio; BMI—body mass index; HDL—high-density lipoprotein; SD—standard deviation.

Subgroup analyses were further performed based on stratifications of age, sex, qualifications, smoking status, physical activity, BMI, inflammatory disease, and metabolic syndrome as well as its components (Figure 3). The relationship between grip strength, muscle mass, and incident CAD remained significant in each subgroup, although modifications by several risk factors were observed. Interactions were noted between grip strength and sex, age, smoking status, physical activity, BMI, inflammatory diseases, and metabolic syndrome. Regarding muscle mass, interactions were observed in age, sex, smoking status, inflammatory disease, and metabolic syndrome. The relationship between grip strength, muscle mass, and incident CAD continued in a dose–response pattern within each subgroup (Figure 4). Non-linear associations were identified between grip strength and incident CAD in subgroups stratified by sex (non-linear *p* = 0.0017 for female and non-linear *p* = 0.0032 for male), age (non-linear *p* = 0.0009 for age < 60 years), qualification (non-linear *p* < 0.0001 for those without college degree), smoking status (non-linear *p* = 0.0002 for previous or current smokers), physical activity (non-linear *p* < 0.0001 for metabolic equivalent [MET] ≥ 500 and non-linear *p* = 0.0055 for MET < 500 min/week), BMI (non-linear *p* = 0.0055 for BMI ≥ 25 kg/m^2^ and non-linear *p* = 0.0001 for BMI < 25 kg/m^2^), inflammatory diseases (non-linear *p* = 0.0005 for those without inflammatory diseases and non-linear *p* = 0.0045 for those with inflammatory diseases), and metabolic syndrome (non-linear *p* < 0.0001 for those without metabolic syndrome and non-linear *p* = 0.0001 for those with metabolic syndrome). As to muscle mass, non-linear relationships were identified in subgroups based on sex (non-linear *p* = 0.0002 for female and non-linear *p* = 0.0173 for male), age (non-linear *p* = 0.0438 for age < 60 years), and metabolic syndrome (non-linear *p* = 0.0001 for those with metabolic syndrome).

We further assessed the potential modifying effect of genetic predisposition in the relationship between physical capability markers and incident CAD. Increased PRS was related with elevated risk of CAD (HR: 1.39; 95% CI 1.38–1.41) (Table S2). Compared to individuals with the lowest PRS, those with intermediate (HR: 1.46; 95% CI 1.42–1.50) and highest PRS (HR: 2.22; 95% CI 2.16–2.29) had higher CAD risk (Table S2). We further found that participants with higher grip strength were associated with a dose–response decreased risk of CAD across all PRS categories (Table S3). Similar trends were observed in participants with higher muscle mass. Furthermore, the association of grip strength and muscle mass with CAD risk was significantly modified by genetic risk (*p*-interaction < 0.001 for grip strength and *p*-interaction = 0.010 for muscle mass).

## 4. Discussion

In this study, we demonstrated that two physical capability markers for sarcopenia, grip strength and muscle mass, were negatively associated with the risk of CAD. Importantly, this association remained significant in participants with potential modifying risk factors including genetic predisposition. Gait speed was also related to the risk of CAD. Grip strength exhibited a non-linear relationship with CAD risk, while linear association was observed between muscle mass and incident CAD. These findings indicated that enhancing muscle strength and muscle mass could potentially serve as protective methods against CAD in middle-aged and elderly people. As for risk assessment, the inclusion of grip strength and/or usual walking pace in existing risk scores has been shown to improve CVD risk prediction [35]. Therefore, considering the potential benefits of incorporating muscle mass, it may be prudent to include this variable in the current risk score equation for CAD.

Several studies conducted using the UK Biobank have previously examined the association between sarcopenia and CVD. For instance, Fanny Petermann-Rocha reported a link between sarcopenia and CVD [36,37]. However, they categorized sarcopenia or physical capability markers and focused on composite CVD outcomes rather than CAD. Thomas Yates demonstrated a negative relationship between self-reported walking pace and the risk of all-cause and cardiovascular mortality, while handgrip strength was associated with cardiovascular mortality in men only [38]. However, they did not investigate the relationship between physical capability markers and the incidence of CAD. Another observational study found an inverse association between absolute grip strength and incident CAD [21], but the shape of this relationship (linear or non-linear) and the interactions between grip strength and other risk factors remained unknown. In individuals with diabetes, those with high grip strength had a lower risk of CVD incidence and mortality. However, this study treated grip strength as a categorical variable and did not explore the independent association between grip strength and CAD [39]. Muscle mass has also been revealed to be associated with the risk of all-cause and CVD mortality, but evidence regarding its relationship with incident CAD was lacking [40,41]. Our study addressed these gaps in the previous literature. A sex-specific population-based cohort study involving one million Swedish men demonstrated that grip strength in early adulthood was a significant predictor of CAD risk later in life, which aligned with our findings in middle-aged and elderly individuals in the UK [42].

To our knowledge, this is the first study analyzing the relationship between physical capability markers and the risk of incident CAD in individuals with different genetic risks. As anticipated, individuals possessing an elevated genetic susceptibility to CAD were at a heightened likelihood of developing CAD. Nevertheless, despite the occurrence of interactions, the dose–response relationship between grip strength, muscle mass, and incident CAD did not manifest any noteworthy alteration depending on whether one possessed a low, intermediate, or high genetic inclination towards CAD. This implies that enhancing muscle mass and quality may prove advantageous in mitigating the risk of CAD, irrespective of genetic predisposition.

This study’s findings hold significant multidisciplinary implications, supporting the integration of muscle mass and strength assessments into cardiovascular screening to facilitate early sarcopenia detection, targeted interventions, reduced CAD risk, and improved quality of life. In public health, they can drive community-based initiatives promoting physical activity and dietary changes among high-risk populations. For research, the findings provide a basis for exploring new therapies and understanding the genetic–environmental–lifestyle interactions in the sarcopenia–CAD relationship, advancing personalized prevention strategies.

It is important to note that our study cannot establish a causal relationship between sarcopenia and CAD risk. The observed association could be confounded by various factors, such as genetic predisposition, socioeconomic status, and lifestyle factors, which were not fully accounted for in our analysis. Future research should consider using more sophisticated study designs, such as Mendelian randomization or longitudinal cohort studies with detailed covariate adjustment, to further investigate the causal nature of this relationship.

Sarcopenia is often associated with a decline in physical function and mobility, which may lead to a more sedentary lifestyle. Reduced physical activity is a well-known risk factor for CAD, as it can contribute to the development of obesity, hypertension, dyslipidemia, and insulin resistance, all of which are major risk factors for CAD. Therefore, the observed association between sarcopenia and CAD risk could be partly explained by the mediating effect of physical inactivity.

The mechanisms elucidating the impact of muscle mass and strength on the development of CAD remain partially understood. Muscle tissue, encompassing both skeletal muscle and myocardium, plays a pivotal role in systemic metabolism [43]. It accounts for approximately 30% of resting energy expenditure and 100% of elevated energy consumption during physical activity [44]. Deterioration in muscle strength or mass leads to reduced physical capacity, consequently contributing to excessive energy accumulation. Recent research has revealed that sarcopenic individuals rely more on carbohydrates rather than fatty acids for energy due to impaired mitochondrial function [45]. Impaired mitochondrial function contributes to ectopic lipid buildup and insulin resistance (IR) [46]. Additionally, skeletal muscle is recognized as an endocrine organ, producing various molecules known as myokines [47]. Numerous myokines, including myostatin, decorin, interleukin-6 (IL-6), Irisin, interleukin-15 (IL-15), meteorin-like protein (Metrnl), and beta-aminoisobutyric acid, have been found to participate in energy metabolism [48]. For instance, irisin, which is decreased in older patients, contributes to chronic inflammation and IR [49,50,51]. Metabolic disorders, inflammations, and IR are well-established risk factors for CVD [7], shedding light on the link between sarcopenia and CAD. In addition to regulating traditional risk factors, reduced muscle quality could impair exercise-induced cardiovascular benefits as a result of compromised physical capacity. Regular exercise modulates the gut microbiota towards a healthier phenotype, potentially decreasing the risk of CVD through microbial metabolite modulation [52,53]. Furthermore, repetitive hemodynamic stimuli during exercise promote antiatherogenic adaptations that enhance vascular endothelial function, promote enlargement of coronary and peripheral vessels, stabilize atherosclerotic plaques, and foster the development of collateral blood vessels [54]. Moreover, sarcopenia is more prevalent in patients with cardiovascular disease than in age-matched controls [55,56], indicating an interaction between skeletal muscle and CAD. Further studies are required to investigate the mechanisms underpinning this interplay and their implications for preventing and managing CAD.

Still, this study has several limitations. Firstly, the data obtained from the UK Biobank represents only a subset of the UK population, and the presence of healthy volunteer bias cannot be disregarded. This limits the generalizability of the findings, including the prevalence and incidence of diseases. Secondly, the measurement of muscle mass relied on bioelectrical impedance analysis (BIA) due to limited data availability for dual-energy X-ray (DXA), which is considered a more accurate method. Although previous research has shown that BIA measurements align with DXA results [26,36], the accuracy of muscle mass might still be affected by factors such as hydration status, food intake, and skin temperature. Thirdly, despite efforts to control for confounding factors, there may be unidentified or residual confounding variables that could have influenced the outcomes. Fourthly, this study utilized walking space as a proxy for gait speed, rather than directly measuring gait speed. Lastly, the observational nature of this study prevents the inference of a causal relationship between grip strength, muscle mass, and CAD.

## 5. Conclusions

Taken together, this study suggested a dose–response and independent association between grip strength, muscle mass, and risk of CAD in middle-aged and elderly individuals enrolled in the UK biobank. Considering global population growth and aging, the prevalence of sarcopenia might increase as well. General assessment of grip strength and muscle mass help to identify individuals at high risk for CAD. Strategies aimed at improving muscle quality may have potential benefits in reducing CAD risk, but more evidence is required to establish causality and inform clinical interventions.

## Figures and Tables

**Table 1:** Baseline characteristics by grip strength and muscle mass in the UK Biobank cohort.

Characteristic	Total	Quintiles of Grip Strength					Quintiles of Muscle Mass				
		1 (the Lowest)	2	3	4	5 (the Highest)	1 (the Lowest)	2	3	4	5 (the Highest)
**n**	439,295	87,858	87,859	87,859	87,859	87,860	87,858	87,859	87,859	87,859	87,860
**Sex (male), n (%)**	198,037 (45.1)	39,577 (45.0)	39,475 (44.9)	38,146 (43.4)	42,501 (48.4)	38,338 (43.6)	1552 (1.8)	11,685 (13.3)	39,927 (45.4)	66,063 (75.2)	78,810 (89.7)
**Age, (mean (SD))**	56.73 (8.10)	59.75 (7.41)	58.55 (7.66)	57.12 (7.86)	55.58 (7.92)	52.64 (7.71)	57.82 (7.68)	57.46 (7.86)	56.90 (8.08)	56.39 (8.23)	55.07 (8.35)
**Deprivation, n (%)**											
Least deprived	87,858 (20.0)	15,113 (17.2)	18,485 (21.0)	17,360 (19.8)	18,183 (20.7)	18,717 (21.3)	15,555 (17.7)	17,654 (20.1)	18,096 (20.6)	18,356 (20.9)	18,197 (20.7)
Second least deprived	87,859 (20.0)	15,989 (18.2)	18,856 (21.5)	17,200 (19.6)	17,998 (20.5)	17,816 (20.3)	16,433 (18.7)	17,911 (20.4)	17,794 (20.3)	17,909 (20.4)	17,812 (20.3)
Medium deprived	87,859 (20.0)	17,009 (19.4)	16,541 (18.8)	19,414 (22.1)	17,385 (19.8)	17,510 (19.9)	17,220 (19.6)	18,145 (20.7)	17,646 (20.1)	17,766 (20.2)	17,082 (19.4)
Second most deprived	87,859 (20.0)	18,153 (20.7)	16,797 (19.1)	17,925 (20.4)	17,592 (20.0)	17,392 (19.8)	18,278 (20.8)	17,548 (20.0)	17,370 (19.8)	17,270 (19.7)	17,393 (19.8)
Most deprived	87,860 (20.0)	21,594 (24.6)	17,180 (19.6)	15,960 (18.2)	16,701 (19.0)	16,425 (18.7)	20,372 (23.2)	16,601 (18.9)	16,953 (19.3)	16,558 (18.8)	17,376 (19.8)
**Qualifications (College or University degree), n (%)**	293,762 (66.9)	64,299 (73.2)	60,701 (69.1)	58,932 (67.1)	56,281 (64.1)	53,549 (60.9)	66,014 (75.1)	61,813 (70.4)	59,099 (67.3)	56,282 (64.1)	50,554 (57.5)
**Ethnicity, n (%)**											
White	415,290 (94.5)	80,828 (92.0)	83,257 (94.8)	83,783 (95.4)	83,874 (95.5)	83,548 (95.1)	82,279 (93.6)	83,503 (95.0)	83,273 (94.8)	83,029 (94.5)	83,206 (94.7)
Mixed	7793 (1.8)	1907 (2.2)	1486 (1.7)	1401 (1.6)	1492 (1.7)	1507 (1.7)	1678 (1.9)	1470 (1.7)	1567 (1.8)	1487 (1.7)	1591 (1.8)
South Asian	7991 (1.8)	3512 (4.0)	1759 (2.0)	1230 (1.4)	919 (1.0)	571 (0.6)	1612 (1.8)	1532 (1.7)	1643 (1.9)	1779 (2.0)	1425 (1.6)
Black	6852 (1.6)	1231 (1.4)	1066 (1.2)	1162 (1.3)	1349 (1.5)	2044 (2.3)	2237 (2.5)	1165 (1.3)	1091 (1.2)	1206 (1.4)	1153 (1.3)
Chinese	1369 (0.3)	380 (0.4)	291 (0.3)	283 (0.3)	225 (0.3)	190 (0.2)	52 (0.1)	189 (0.2)	285 (0.3)	358 (0.4)	485 (0.6)
**Smoking status, n (%)**											
Never	243,950 (55.5)	48,447 (55.1)	48,722 (55.5)	48,768 (55.5)	48,533 (55.2)	49,480 (56.3)	50,964 (58.0)	50,352 (57.3)	46,809 (53.3)	46,227 (52.6)	49,598 (56.5)
Previous	149,514 (34.0)	30,198 (34.4)	30,509 (34.7)	30,236 (34.4)	29,932 (34.1)	28,639 (32.6)	29,775 (33.9)	29,607 (33.7)	31,957 (36.4)	31,803 (36.2)	26,372 (30.0)
Current	45,831 (10.4)	9213 (10.5)	8628 (9.8)	8855 (10.1)	9394 (10.7)	9741 (11.1)	7119 (8.1)	7900 (9.0)	9093 (10.3)	9829 (11.2)	11,890 (13.5)
**Diet score, n (%)**											
Higher quintile	9945 (2.3)	1836 (2.1)	2007 (2.3)	2022 (2.3)	2023 (2.3)	2057 (2.3)	2161 (2.5)	2298 (2.6)	1975 (2.2)	1546 (1.8)	1965 (2.2)
4th quintile	142,711 (32.5)	27,624 (31.4)	29,197 (33.2)	29,272 (33.3)	28,472 (32.4)	28,146 (32.0)	30,194 (34.4)	31,760 (36.1)	27,579 (31.4)	26,141 (29.8)	27,037 (30.8)
3rd quintile	212,420 (48.4)	42,320 (48.2)	42,007 (47.8)	42,313 (48.2)	42,857 (48.8)	42,923 (48.9)	43,147 (49.1)	41,779 (47.6)	42,633 (48.5)	43,000 (48.9)	41,861 (47.6)
2nd quintile	70,070 (16.0)	14,998 (17.1)	13,858 (15.8)	13,474 (15.3)	13,754 (15.7)	13,986 (15.9)	11,834 (13.5)	11,473 (13.1)	14,854 (16.9)	16,143 (18.4)	15,766 (17.9)
Lower quintile	4149 (0.9)	1080 (1.2)	790 (0.9)	778 (0.9)	753 (0.9)	748 (0.9)	522 (0.6)	549 (0.6)	818 (0.9)	1029 (1.2)	1231 (1.4)
**Physical activity, MET-min/week (%)**											
<500	147,502 (33.6)	35,273 (40.1)	30,642 (34.9)	29,015 (33.0)	27,026 (30.8)	25,546 (29.1)	38,747 (44.1)	31,553 (35.9)	28,845 (32.8)	25,906 (29.5)	22,451 (25.6)
≥500	291,793 (66.4)	52,585 (59.9)	57,217 (65.1)	58,844 (67.0)	60,833 (69.2)	62,314 (70.9)	49,111 (55.9)	56,306 (64.1)	59,014 (67.2)	61,953 (70.5)	65,409 (74.4)
**BMI, n (%)**											
<25	148,472 (33.8)	28,231 (32.1)	30,673 (34.9)	30,854 (35.1)	29,850 (34.0)	28,864 (32.9)	1907 (2.2)	26,506 (30.2)	39,458 (44.9)	28,702 (32.7)	51,899 (59.1)
≥25	290,823 (66.2)	59,627 (67.9)	57,186 (65.1)	57,005 (64.9)	58,009 (66.0)	58,996 (67.1)	85,951 (97.8)	61,353 (69.8)	48,401 (55.1)	59,157 (67.3)	35,961 (40.9)
**Inflammatory diseases (yes), n (%)**	65,579 (14.9)	16,046 (18.3)	12,906 (14.7)	12,445 (14.2)	12,122 (13.8)	12,060 (13.7)	17,043 (19.4)	13,499 (15.4)	12,287 (14.0)	11,755 (13.4)	10,995 (12.5)
**Metabolic syndrome (yes), n (%)**	127,711 (29.1)	30,621 (34.9)	25,879 (29.5)	24,513 (27.9)	23,771 (27.1)	22,927 (26.1)	47,137 (53.7)	24,224 (27.6)	26,245 (29.9)	20,731 (23.6)	9374 (10.7)
**Central obesity (yes), n (%)**	131,167 (29.9)	30,361 (34.6)	26,086 (29.7)	25,157 (28.6)	24,445 (27.8)	25,118 (28.6)	64,603 (73.5)	25,272 (28.8)	25,649 (29.2)	13,720 (15.6)	1923 (2.2)
**High glycaemia/diabetes (yes), n (%)**	75,651 (17.2)	19,809 (22.5)	15,997 (18.2)	14,665 (16.7)	13,529 (15.4)	11,651 (13.3)	19,708 (22.4)	15,129 (17.2)	16,030 (18.2)	14,340 (16.3)	10,444 (11.9)
**High blood pressure/hypertension (yes), n (%)**	324,139 (73.8)	67,928 (77.3)	66,157 (75.3)	64,802 (73.8)	63,806 (72.6)	61,446 (69.9)	70,697 (80.5)	62,395 (71.0)	63,377 (72.1)	66,476 (75.7)	61,194 (69.6)
**High triglycerides (yes), n (%)**	165,529 (37.7)	36,014 (41.0)	33,707 (38.4)	32,683 (37.2)	32,599 (37.1)	30,526 (34.7)	38,602 (43.9)	30,011 (34.2)	32,522 (37.0)	36,810 (41.9)	27,584 (31.4)
**Low HDL (yes), n (%)**	130,319 (29.7)	28,100 (32.0)	25,447 (29.0)	25,301 (28.8)	25,318 (28.8)	26,153 (29.8)	37,105 (42.2)	27,688 (31.5)	24,585 (28.0)	22,690 (25.8)	18,251 (20.8)

Descriptive characteristics by quintile of grip strength are presented as means with SD for quantitative variables and as frequencies and percentages for categorical variables. MET—metabolic equivalent; BMI—body mass index; HDL—high-density lipoprotein; n—number; SD—standard deviation.

## Data Availability

The datasets used and analyzed during the current study are available on application to the UK Biobank (www.ukbiobank.ac.uk/). This research has been conducted using the UK Biobank Resource under application number 109546.

## Supplementary Materials

The following supporting information can be downloaded at: https://www.mdpi.com/article/10.3390/healthcare13091018/s1, Figure S1: Flow diagram participants included in the study; Figure S2: Directed acyclic graph (DAG) explaining the association between the exposures, the outcome, and covariates included in the analyses; Figure S3: Association between grip strength, muscle mass, and coronary heart disease (Excluding people with extreme valves); Figure S4: Associations between categories of gait speed (walking pace) and coronary heart disease; Table S1: Definitions and sources of information for outcomes in UK Biobank; Table S2: Multivariable-adjusted HRs (95%CIs) for PRS, PRS quartiles, and CAD; Table S3: Association of grip strength, muscle mass, and CAD by genetic predisposition.
