# Supplementary material for: Associations Between Physical Capability Markers and Risk of Coronary Artery Disease: A Prospective Study of 439,295 UK Biobank Participants
Source: Healthcare (Basel). 2025 Apr 28;13(9):1018. doi: 10.3390/healthcare13091018 (PMC12071247; doi:10.3390/healthcare13091018)
Supplement: Supplementary file 1 [file healthcare-13-01018-s001.zip › healthcare-3502106-supplementary.pdf]

Supplementary Materials

1. Supplementary Figures

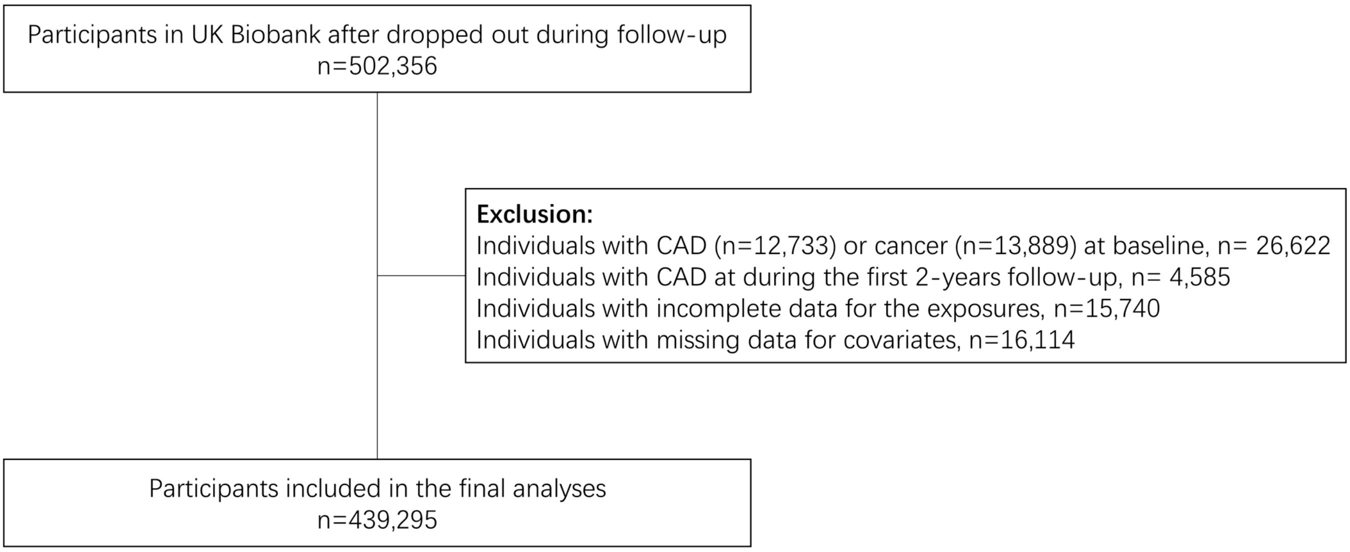

Figure S1. Flow diagram participants included in the study.

UK, United Kingdom; CAD, coronary artery disease.

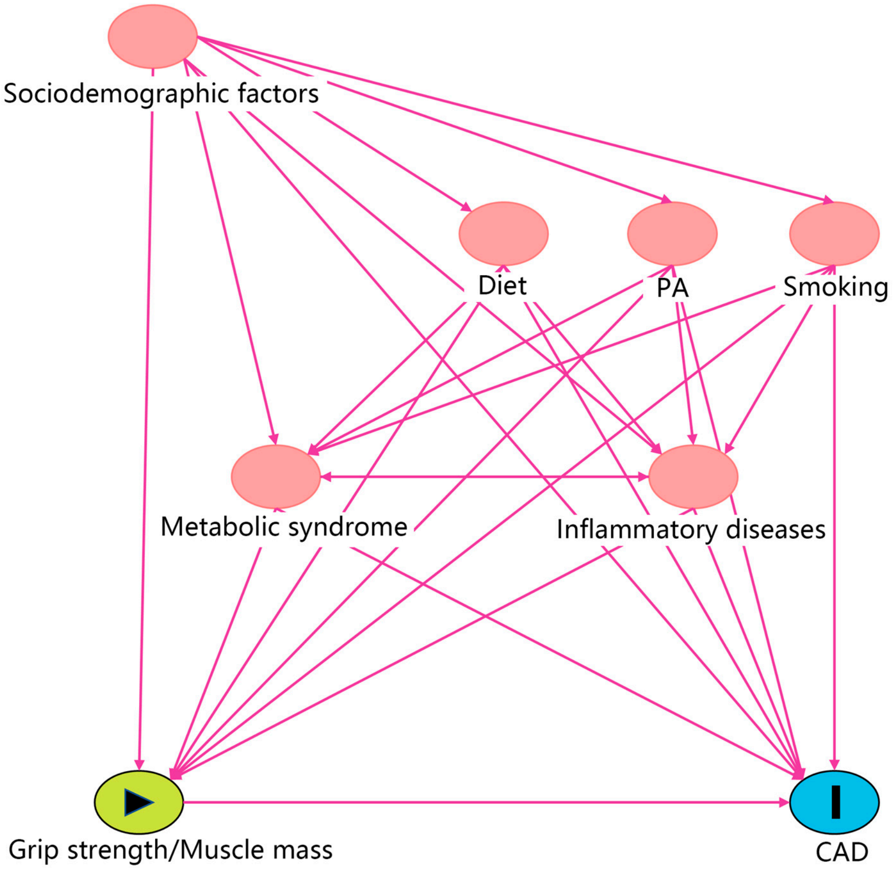

Figure S2. Directed acyclic graph (DAG) explaining the association between the exposures, the outcome, and covariates included in the analyses.

DAG was drawn using http://www.dagitty.net/. Sociodemographic factors encompassed age, sex, deprivation and ethnicity; inflammatory disease included arthritis, inflammatory bowel disease and asthma; metabolic syndromes include central obesity, high blood pressure/hypertension, diabetes/high glucose levels, high triglyceride concentrations and low HDL. PA: physical activity; HDL, high-density lipoprotein.

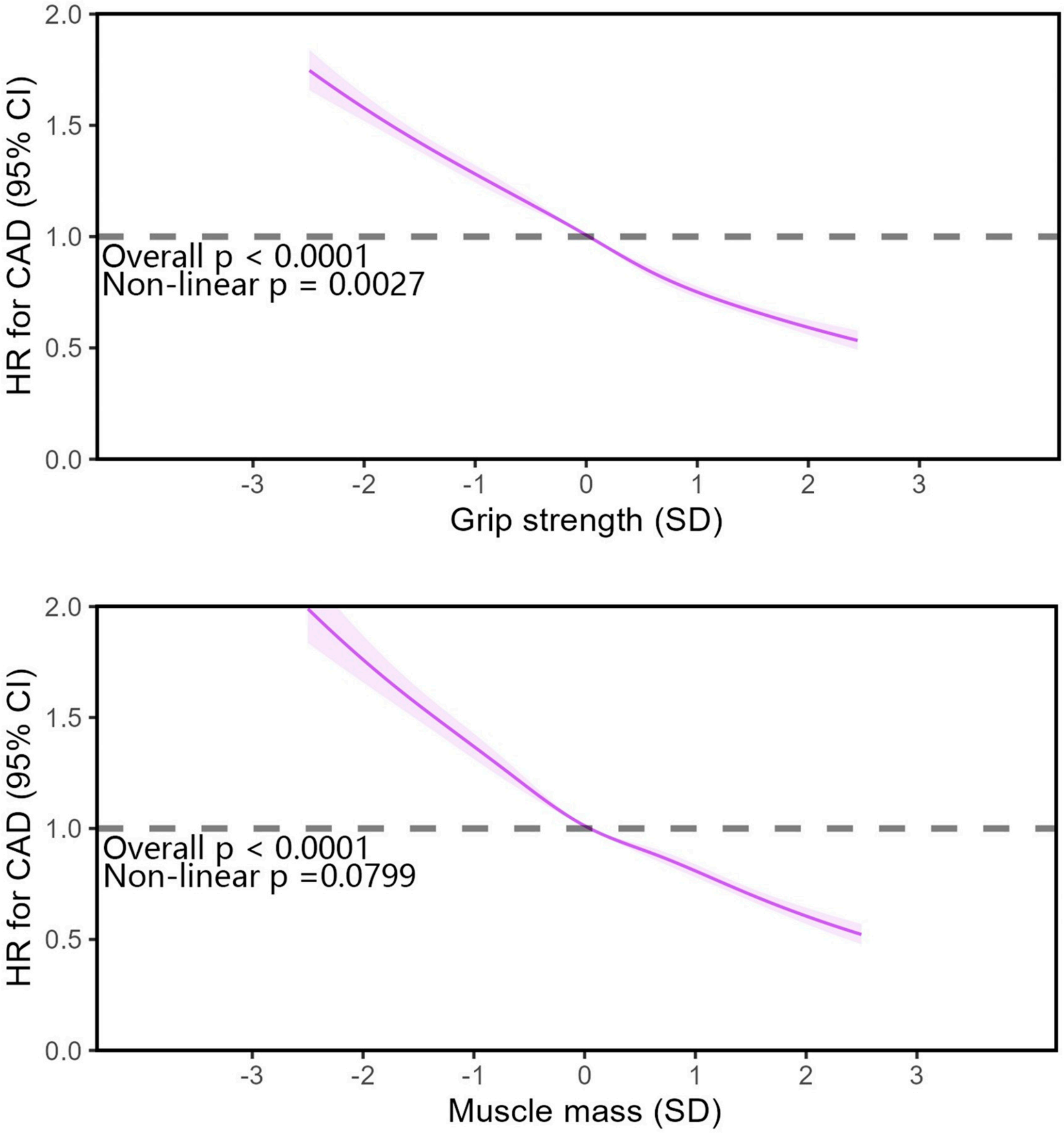

Figure S3. Association between grip strength, muscle mass and coronary heart disease (Excluding people with extreme valves)

The non-linear relationships between grip strength, muscle mass, and CAD were analyzed by employing penalized cubic splines in Cox proportional hazard models. The average value of each exposure was selected as the reference group. Participants with existing CAD or cancer at baseline, or with insufficient data on the exposures or covariates, were excluded from the analyses. Potential confounders including age, sex, qualification, deprivation, ethnicity, smoking, physical activity, diet score, inflammatory disease, BMI and metabolic syndrome (central obesity, high glycaemia/diabetes, high blood pressure/hypertension, low HDL, and high triglyceride) were adjusted in the analysis. A p value $<$ 0.05 was considered statistically significant. CAD, Coronary artery disease; HR, hazard ratio; BMI, body mass index; HDL, high-density lipoprotein; SD, standard deviation.

	N	Events	HR(95% CI)	P-value
**Gait speed**				
Slow pace	31098	6259 (20.1)	1.00 (Ref.)	
Average pace	231574	22751 (9.8)	0.54 [0.52, 0.55]	< 0.001
Brisk pace	176623	11046 (6.3)	0.39 [0.38, 0.41]	< 0.001

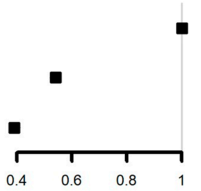

Figure S4. Associations between categories of gait speed (walking pace) and coronary heart disease.
Associations between gait speed (walking pace) and CAD were analyzed using Cox proportional hazard models. Participants with CAD at baseline or missing data for exposures or covariates were excluded from the analyses. Participants with existing CAD or cancer at baseline, or with insufficient data on the exposures or covariates, were excluded from the analyses. Potential confounders including age, sex, qualification, deprivation, ethnicity, smoking, physical activity, diet score, inflammatory disease, BMI and metabolic syndrome (central obesity, high glycaemia/diabetes, high blood pressure/hypertension, low HDL, and high triglyceride) were adjusted in the analysis. A p value < 0.05 was considered statistically significant. CAD, Coronary artery disease; N, number; HR, hazard ratio; BMI, body mass index; HDL, high-density lipoprotein; SD, standard deviation.

2. Supplementary Tables

Table S1. Definitions and sources of information for outcomes in UK Biobank

	Number of cases	ICD-9 diagnosis	ICD-10 diagnosis	OPCS procedure	Self-report[a]
Coronary artery disease	40 056	410.X, 411.X, 412.X, 414.0, 414.8, 414.9	I20.X,I21.X, I22.X, I23.X, I24.X, I25.1, I25.2, I25.3,I25.4,I25.5, I25.6, I25.8, I25.9	K40.X, K41.X, K42.X, K43.X, K44.X, K45.X, K46.X, K49.X, K50.1, K50.2, K50.4, K75.X	6150, 20002, 20004

Abbreviations: ICD, International Classification of Disease; OPCS, Office of Population Censuses and Surveys Classification of Surgical Operations and Procedures.

[a]Numbers refer to data codes used in UK Biobank: 6150 = Health condition diagnosed by doctor (self-reported from touchscreen); 20002 = non-cancer illness code (self-reported from interview with nurse); 20004 = Surgical operation code (self-reported from interview with nurse).

Table S2. Multivariable-adjusted HRs (95%CIs) for PRS, PRS quartiles and CAD.

	Total cases	Total sample size	HRs (95% CI)
**PRS**	40056 (9.1)	439295	1.39 (1.38-1.41)
**PRS quartiles**			
Low (Q1)	6569 (6.0)	109823	ref
Intermediate (Q2, Q3)	19191 (8.7)	219649	1.46 (1.42-1.50)
High (Q4)	14296 (13.0)	109823	2.22 (2.16-2.29)

Adjusted for sex, age, deprivation, qualifications, ethnicity, smoking status, diet score, physical activity, BMI, inflammatory diseases, and metabolic syndrome (central obesity, high glycaemia/diabetes, high blood pressure/hypertension, low HDL, and high triglyceride). PRS, polygenic risk score; HR, hazard ratio; Q, quartile; BMI, body mass index.

Table S3. Association of grip strength, muscle mass and CAD by genetic predisposition.

	Group	HRs (95% CI)	P for interaction
**Grip strength**	**Low genetic risk**		<0.001
	Quintile 1	1	
	Quintile 2	0.79 (0.73-0.84)	
	Quintile 3	0.71 (0.66-0.76)	
	Quintile 4	0.55 (0.51-0.60)	
	Quintile 5	0.44 (0.40-0.48)	
	**Intermediate genetic risk**		
	Quintile 1	1	
	Quintile 2	0.79 (0.76-0.82)	
	Quintile 3	0.69 (0.66-0.72)	
	Quintile 4	0.62 (0.60-0.65)	
	Quintile 5	0.49 (0.47-0.52)	
	**High genetic risk**		
	Quintile 1	1	
	Quintile 2	0.85 (0.82-0.90)	
	Quintile 3	0.75 (0.72-0.79)	
	Quintile 4	0.63 (0.60-0.66)	
	Quintile 5	0.52 (0.49-0.55)	
**Muscle mass**	**Low genetic risk**		0.010
	Quintile 1	1	
	Quintile 2	0.84 (0.77-0.92)	
	Quintile 3	0.69 (0.62-0.76)	
	Quintile 4	0.60 (0.53-0.68)	
	Quintile 5	0.52 (0.45-0.61)	
	**Intermediate genetic risk**		
	Quintile 1	1	
	Quintile 2	0.83 (0.79-0.88)	
	Quintile 3	0.69 (0.64-0.73)	
	Quintile 4	0.64 (0.60-0.69)	
	Quintile 5	0.53 (0.49-0.58)	
	**High genetic risk**		
	Quintile 1	1	
	Quintile 2	0.82 (0.77-0.87)	
	Quintile 3	0.71 (0.66-0.76)	
	Quintile 4	0.66 (0.61-0.72)	
	Quintile 5	0.55 (0.50-0.61)	

Adjusted for sex, age, deprivation, qualifications, ethnicity, smoking status, diet score, physical activity, BMI, inflammatory diseases, and metabolic syndrome (central obesity, high glycaemia/diabetes, high blood pressure/hypertension, low HDL, and high triglyceride). HR, hazard ratio; BMI, body mass index.
